# Why are pediatricians uncomfortable with prescribing emergency contraception for adolescents?

**DOI:** 10.1590/1984-0462/2023/41/2022060

**Published:** 2023-05-29

**Authors:** Renata Vieira Amorim, Marco Antônio Barbieri, Camila Bôtto-Menezes, Fábio Carmona, Alexandre Archanjo Ferraro, Heloisa Bettiol

**Affiliations:** a Universidade do Estado do Amazonas, Manaus, AM, Brazil.; b Universidade de São Paulo, Ribeirão Preto, SP, Brazil.; c Fundação de Medicina Tropical Doutor Heitor Vieira Dourado, Manaus, AM, Brazil.; d Universidade de São Paulo, São Paulo, SP, Brazil.

**Keywords:** Contraception, postcoital, Pregnancy in adolescence, Pregnancy, unplanned, Pediatricians, Artificial intelligence, Anticoncepção pós-coito, Gravidez na adolescência, Gravidez não planejada, Pediatras, Inteligência artificial

## Abstract

**Objective:**

Emergency contraception (EC) is an effective and safe method for preventing unplanned pregnancy after unprotected sexual intercourse among adolescents but is infrequently prescribed by pediatricians. Because of the scarcity of data on the discomfort with EC prescription among physicians in Brazil, this study aimed to identify associated factors with discomfort with EC prescription among pediatricians in the state of Amazonas.

**Methods:**

A web-based, cross-sectional study including sociodemographic data, knowledge, attitudes, and discomfort with EC prescription was used. Multivariate logistic regression and artificial intelligence methods such as decision tree and random forest analysis were used to identify factors associated with discomfort with EC prescriptions.

**Results:**

Among 151 physicians who responded to the survey, 53.0% were uncomfortable with prescribing EC, whereas only 33.1% had already prescribed it. Inexperience was significantly associated with discomfort with EC prescription (odds ratio 4.47, 95% confidence interval 1.71–11.66). Previous EC prescription was protective against discomfort with EC prescription in the three models.

**Conclusions:**

EC is still infrequently prescribed by pediatricians because of inexperience and misconceptions. Training these professionals needs to be implemented as part of public health policies to reduce unplanned adolescent pregnancy.

## RESUMO

**Objetivo:**

A contracepção de emergência (CE) é um método eficaz e seguro para prevenir gravidez não planejada após relação sexual desprotegida entre adolescentes, mas raramente prescrito por pediatras. Diante da escassez de dados sobre o desconforto com a prescrição de CE entre médicos no Brasil, o objetivo deste estudo foi identificar fatores associados a esse desconforto entre pediatras do estado do Amazonas.

**Métodos:**

Uma pesquisa do tipo *e-survey* coletou dados sociodemográficos, conhecimento, atitudes e desconforto com relação à prescrição de CE. Métodos de regressão logística multivariada e inteligência artificial, como árvore de decisão e *random forest*, foram usados para identificar fatores associados ao desconforto para a prescrição de CE.

**Resultados:**

Entre os 151 médicos que responderam à pesquisa, 53,0% sentiam-se desconfortáveis para prescrever CE e apenas 33,1% já a haviam prescrito. A inexperiência foi associada a esse desconforto (*odds ratio* — OR 4,47, intervalo de confiança — IC95% 1,71–11,66). A prescrição prévia de CE foi fator de proteção com relação ao desconforto nos três modelos.

**Conclusões:**

A CE ainda é pouco prescrita por pediatras. Apesar de sua segurança e eficácia, a inexperiência e conceitos equivocados foram associados ao desconforto para sua prescrição. Investigações sobre o assunto são importantes para subsidiar políticas públicas de saúde para a redução da gravidez não intencional na adolescência.

## INTRODUCTION

Emergency contraception (EC) or postcoital contraception is a method to prevent pregnancy after unprotected sexual intercourse in the absence or failure of other contraceptive methods.^1^ EC is not a first-choice method but offers a second chance to avoid unplanned pregnancy.^2^

Currently, levonorgestrel (LNG) is the drug of choice because it is efficacious and causes fewer adverse effects.^3,4,5^ LNG is available in about 150 countries.^6^ It reduces the risk of pregnancy if used up to 120 h after the sexual intercourse, with greater efficacy if used in the first 24 h.^2,6^ LNG delays ovulation but does not affect egg fertilization or embryo implantation.^6–8^ No associations were found between LNG and teratogenic outcomes, abortion, or tubal pregnancy.^8,9,10^ LNG is effective, safe, and recommended for contraception in adolescents.^1^

EC is essential for adolescents because they tend to have more unplanned sexual intercourses, low adherence to contraceptive methods, and misuse due to inexperience.^7,11^ Adolescents living in the Northern regions of Brazil are more vulnerable to unprotected sexual intercourse outcomes.^12^ About 50–80% of teenage pregnancies are unintentional.^7,13^ Unplanned adolescent pregnancy is a public health issue that transcends health complications, with its socioeconomic impact including a high school dropout rate, lower qualifications, and poorer employment status.^14^

Despite being approved in Brazil since 1999, and being effective, affordable, and safe in adolescents, EC is still little known and infrequently prescribed by Brazilian pediatricians.^15^ The reasons why they do not prescribe EC for adolescents are unknown to the best of our knowledge.

Therefore, this study aimed to investigate the factors associated with discomfort for EC prescription among pediatricians from the Amazonas state, Brazil.

## METHOD

This cross-sectional observational study (online survey) was planned to investigate the factors associated with discomfort with EC prescription among physicians assisting adolescents. The Ethics Committee of the Amazonas State University (UEA) approved this study under n. 2.486.595/18. All participants filled out an electronic informed consent form before taking the survey.

The population of interest consisted of physicians working with pediatrics in Amazonas, Brazil. All physicians who meet this criterion (n=808) were invited to participate in the study by e-mail. Their electronic addresses were obtained from the following institutions: Regional Council of Medicine of Amazonas State, Amazonian Pediatric Society, Amazonas State Health Department, Manaus Municipal Health Department, and private clinics that provide pediatric services to the public health system.

A convenience sample consisting of 151 respondents was used. If we assume a frequency of discomfort with EC prescription of 51%,^15^ a 5% probability of occurring type I error, and a statistical power of 80%, a sample of 140 respondents would be able to identify an association, as long any given risk factor had frequencies of 30 and 15% in the groups with positive and negative outcomes, respectively.

An anonymous, structured electronic questionnaire with 25 items was used to collect sociodemographic data, knowledge, previous experience with EC, and perception of discomfort with prescribing EC among the participants. The questionnaire was based on previous studies.^15–17^ The survey was conducted using REDCap (Research Electronic Data Capture, https://project-redcap.org).

Recruitment and data collection occurred from March 14 to June 19, 2018. Data collection and storage followed international confidentiality standards for health research.^18^

Multivariate logistic regression, decision tree, and random forest methods were used to assess factors associated with discomfort with EC prescription.

Statistical analysis was performed using the R 3.2.5 software, whereas the C5.0 and Caret packages were used for decision tree and random forest analyses, respectively.

The decision tree is an artificial intelligence method used in machine learning and data mining; its scope is to discover the predictive structure of a problem.^19^ In the present study, the decision tree identified valid standards and relationships correlating variables with discomfort for EC prescription.

In random forest analysis, the original data set was divided into smaller subsets with randomly selected variables. Hence, each subset originated a different decision tree. In this analysis, the final model considered the prediction of all trees, yielding better predictive performance.^20^

K-fold cross-validation was used to test the models’ performances in our study, consisting of two phases, namely, training and validation.^19^ Briefly, our database was split into five equal or almost equal subsets (k=5). The first subset was the validation set, and the others were training subsets. The training and validation sets were crossed over in successive rounds.

## RESULTS

**Figure 1.**
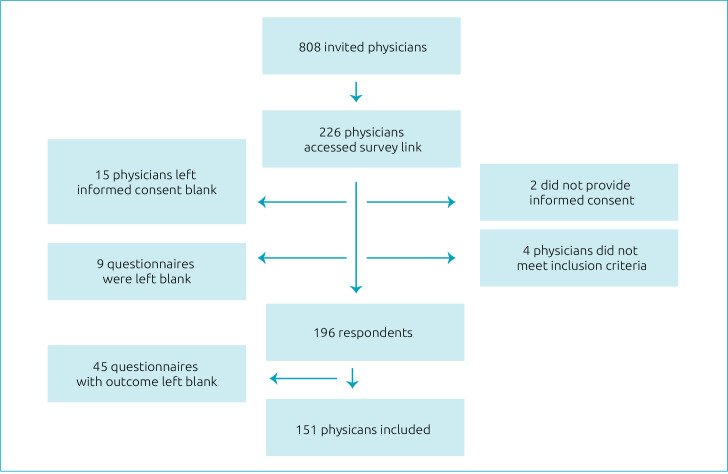
Study flowchart.

Of the 808 invited physicians, 196 responded to the questionnaire (response rate of 24.3%). Also, 45 participants did not respond to the question regarding discomfort with EC prescription and therefore were excluded. Thus, 151 participants were included in the analysis (Figure 1).

The characteristics of the participants are presented in Table 1. Most professionals (53.0%) reported discomfort with EC prescriptions. The mean age was 40.5 (±9.8) years, and women were predominant (80.1%). The mean time of experience in pediatrics was 13.8 (±9.9) years. Notably, 61% of the participants attended medical residency in pediatrics, 76.2% cared for adolescents in their offices, and the average number of adolescents seen per month was 35.6 (±75.6). The most frequent practice settings were public hospitals (72.8%) and public outpatient clinics (53.0%).

**Table 1.** Characteristics of physicians working with pediatrics in Amazonas state.

Variables	Mean±SD or n		Range or %
Age in years (n=151)	40.5±9.8		23.0–72.0
Graduation year (n=151)	2003±9.9		1972–2017
Years of experience in pediatrics (n=151)	13.8±9.9		0–45
Number of adolescents seen/month (n=140)	35.6±75.6		0–512
Interest in learning more about EC (n=151)	84.6±1.6		0–100
Gender (n=151)	Female	121	80.1
	Male	30	19.9
Pediatric residency (n=150)	Yes	92	61.3
	No	58	38.7
Practice setting (n=151)*	Public hospital	110	72.8
	Public outpatient clinic	80	53.0
	Private hospital	67	44.4
	Private clinic	66	43.7
	Emergency	62	41.1
Academic activity (n=151)		42	27.8
Working place (n=149)	Capital	145	97.3
	Inland	2	1.3
	Both	2	1.3
Adolescent patients care (n=151)	Yes	115	76.2
	No	36	23.8
Discomfort about EC prescription (n=151)	Yes	80	53.0
	No	71	47.0

EC: emergency contraception; SD: standard deviation. *More than one option possible.

Only 13.9% of physicians were familiar with the EC methods approved in Brazil, while 15.2% knew the maximum time after the intercourse for EC prescription. Around two-thirds of physicians had never prescribed EC before. Less than one-quarter of physicians correctly answered that previous consent of the guardians before EC prescription was unnecessary in most cases (Table 2).

**Table 2.** Knowledge and experience of physicians about emergency contraception.

Variables		n	%
EC method released in Brazil			
EC prescription			
Yuzpe method and LNG (n=151)	Yes	21	13.9
	No	130	86.1
Maximum time (n=132)	24 hours	27	20.5
	72 hours	85	64.4
	120 hours	20	15.2
Physical examination before EC prescription (n=138)	Unnecessary	11	8.0
	Recommended	35	25.4
	Always necessary	92	66.7
Pelvic examination before EC prescription (n=133)	Unnecessary	26	19.5
	Recommended	60	45.1
	Always necessary	47	35.3
Pregnancy test before EC prescription (n=137)	Unnecessary	55	40.1
	Recommended	28	20.4
	Always necessary	54	39.4
Legal guardian consent (n=120)	Unnecessary most of the time	26	21.7
	Recommended	56	46.7
	Always necessary	38	31.7
Experience			
Theoretical learning (n=151)	Yes	105	69.5
	No	46	30.5
Practical learning (n=151)	EC prescription	50	33.1
	Not prescribed	101	66.9
Reasons why prescription was requested (n=50)*			
Unprotected sex		34	68.0
Forgetfulness of pill		19	38.0
Ruptured condom		18	36.0
Sexual violence		15	30.0
Has not prescribed or would not prescribe			
Exceeded the maximum time to prescribe EC (n=122)		77	63.1
Inexperience with use (n=137)		74	54.0
Referral of patient to another colleague (n=129)		53	41.1
The belief that EC compromises adherence to other methods (n=128)		45	35.2
The belief that EC stimulates sexual risk behavior (n=128)		40	31.3
Fear of teratogenic effects (n=131)		36	27.5
Religious reasons (n=131)		21	16.0
Moral reasons (n=129)		19	14.7
The belief that EC causes abortion (n=125)		18	14.4
EC is not effective (n=119)		1	0.8

EC: emergency contraception; LNG: levonorgestrel. *More than one option possible.

Inexperience was an important reason (54.0%) for not prescribing EC. Most professionals (63.1%) did not prescribe EC to patients because they thought the maximum time for its prescription had been exceeded. Moreover, around one-third of physicians avoided EC prescription for the following reasons: belief that it promoted risky sexual behavior, belief that EC would compromise adherence to other contraceptive methods, and fear of teratogenic or abortive effects. Notably, almost half of the participants referred the adolescent to a colleague instead of prescribing EC themselves (Table 2).

Since not every question in the survey was answered, only 111 physicians were included in the multivariate analysis. Having a previous experience with EC prescription was protective against discomfort from its prescription, while inexperience with EC was a risk factor for discomfort with prescribing EC. Likewise, the belief that EC compromises adherence to other contraceptive methods was positively associated with discomfort with prescribing EC. Each unit’s increased interest in learning more about EC reduced discomfort with EC prescription by 4% (Table 3).

**Table 3.** Final multivariate analysis model of factors associated with discomfort with prescribing emergency contraception.

Factors associated with discomfort about EC prescription	OR (95%CI)
Practical experience – previous EC prescription	0.20 (0.06–0.59)
It has not been prescribed due to inexperience with the use	4.47 (1.71–11.66)
It has not been prescribed because of the belief that it compromises adherence to other methods	4.68 (1.64–13.38)
Interest in learning more about EC	0.96 (0.93–0.99)

EC: emergency contraception; OR: odds ratio; 95%CI: 95% confidence interval.

**Figure 2.**
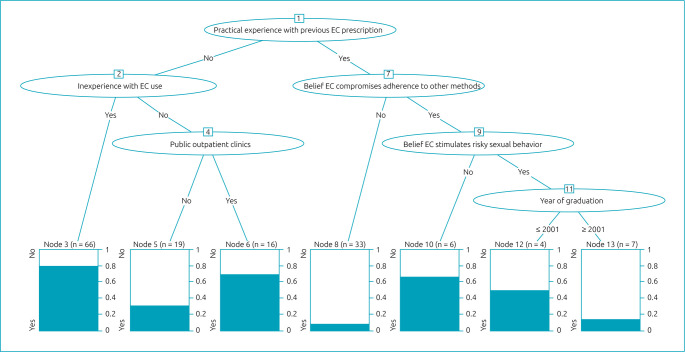
Decision tree for discomfort with emergency contraception prescription. Practical experience with previous emergency contraception prescription was the most relevant variable in the model. Among those without practical experience, factors associated with more discomfort with emergency contraception prescription were inexperience and working in public outpatient clinics. Among those with practical experience, the factors increasing discomfort with emergency contraception prescription were the belief that emergency contraception compromises adherence to other methods and stimulates risky sexual behavior and having graduated before 2001.

The decision tree has a graphical representation of an inverted tree, and all 151 participants were categorized according to their discomfort with EC prescription. Six independent variables were associated with the outcome and represented in ovals, also called inner nodes. Practical experience with previous EC prescription was the root node, corresponding to the most relevant variable in the model (Figure 2). Among those without practical experience, factors associated with more discomfort with EC prescription were inexperience and working in public outpatient clinics. Among those with practical experience, the factors increasing discomfort with EC prescription were the belief that EC compromises adherence to other methods and stimulates risky sexual behavior and having graduated before 2001.

The main result of the random forest was a ranking of the importance of the variables according to their association with discomfort for EC prescription. The most relevant were inexperience with EC, number of adolescents seen per month, no practical experience, and physician’s age. The respective percentages of importance were 100, 73.4, 69.2, and 68.7%. In contrast to the decision tree, the random forest did not offer a cutoff point for continuous variables. In random forest, 127 physicians were included since variables with 10% or more missing data were not considered.

Cross-validation was used to check the model’s performance by calculating sensitivity and specificity. Logistic regression had the highest values of accuracy (0.818), sensitivity (0.864), and specificity (0.770). The random forest had higher accuracy and sensitivity than the decision tree, 0.715 versus 0.649 and 0.809 versus 0.695, respectively. Specificity was higher for the decision tree (0.603) than that for the random forest (0.600).

## DISCUSSION

Although EC has been available for medical prescription since the 1970s, its prescription still causes discomfort for 53% of the pediatricians who work in the state of Amazonas. Previous studies conducted in the city of São Paulo (Brazil) and the New York metropolitan area obtained similar frequencies of discomfort for EC prescription among pediatricians, i.e., 51.1 and 68.4%, respectively.^15,16^

In the present study, discomfort with EC prescription was associated with misconceptions, inexperience with its use, and lack of practical experience.

Most participants reported inexperience as one of the main reasons for not prescribing EC. In the three statistical models, inexperience with EC use was positively associated with discomfort with EC prescription. Other studies have reported inexperience as the most crucial reason for the nonprescription of EC (64–70%) among Brazilian and American pediatricians.^15–17^

A previous practical experience with EC prescription had a protective effect against discomfort with EC prescription in the three models. Even though about 70% of participants reported learning the theory about the subject, this factor was not associated with discomfort for EC prescription. Since previous practical experience with EC reduced the discomfort with its prescription and theoretical learning did not, simulation-based medical education (SBME) might facilitate learning and stimulate the practice of EC prescription. Thus, contraceptive counseling-simulated scenarios could be created with standardized patients.^21^

Exceeding the maximum time for EC prescription was the main reason for not recommending the method. Most participants believed EC should be used up to 72 h after intercourse rather than after 120 h. This fact could have led to EC under prescription. In addition, most physicians were unaware that physical examination, pelvic examination, and pregnancy test were not required before EC prescription.

Only one-fifth of physicians knew that parental consent for EC prescription is also not required in most cases. Ordering tests or asking for parental consent compromises or delay EC use, decreasing its effectiveness in preventing unplanned pregnancy.

No association was found between EC knowledge and discomfort with EC prescription. Nevertheless, knowledge was not associated with discomfort with EC prescription, but a lack of it could lead to the under-use of EC. A cohort study in California (USA) showed that improved knowledge after an educational intervention was associated with higher rates of EC prescription.^22^

Nonprescription of EC due to the belief that it compromises adherence to other methods was associated with discomfort to prescribe EC in logistic regression and decision tree. Goyal et al. reported that one-third of emergency pediatricians considered that EC prescription discouraged using other contraceptive methods.^23^ In the present study, one-third of the participants did not prescribe EC due to fear it could stimulate risky sexual behavior. This belief was associated with discomfort with EC prescription in the decision tree. Previous studies and systematic reviews did not find associations between EC and risky sexual behavior, such as increased frequency of unprotected sex or sexually transmitted infections.^24–26^

One-third of participants avoided EC prescription due to fear of teratogenic effects, although this association was not observed in cohort studies.^10,27^ Some respondents reported religious and moral reasons for not recommending EC. These barriers may be because of unfamiliarity with the mechanism of action of EC, but this study was not designed to answer this question. One-fifth of the gynecologists interviewed in a Brazilian study had misconceptions about the mechanism of action of LNG.^28^

Even though 76.2% of the physicians studied here care for adolescents in their offices, most of them felt uncomfortable prescribing EC. The number of adolescents seen per month was associated with discomfort with EC prescription in the random forest model. Likewise, an American study identified an association between pediatricians’ higher rates of EC prescription and 10 or more adolescents seen per week.^14^

The physician’s age was associated with discomfort for EC prescription in the random forest model. Golden et al. did not find a difference in the comfort level with prescribing EC based on age, gender, or among general pediatricians or subspecialists.^16^

Working at public outpatient clinics was associated with discomfort with EC prescription in the decision tree. On the contrary, Goyal et al. reported that pediatricians trained in emergency medicine had a twice higher chance of prescribing EC.^23^

In the present study, the decision tree model showed that physicians finishing medical school in 2001 or before were more uncomfortable with prescribing EC than those who completed medical school after 2001. Possibly, this was because EC was approved in Brazil in 1999 when medical schools started to train their students on the subject. Attending the pediatric residency program was not associated with discomfort with EC prescription.

Despite the better performance of multivariate logistic regression indicators in cross-validation, our results should be interpreted with caution since 36% of participants were not included in the analysis due to missing data; machine learning methods such as decision tree and random forest deal better with missing data.^20^

The present study has some potential limitations. One is that causality cannot be assumed in a cross-sectional study. The electronic survey was used in an attempt to access pediatricians all over the Amazonas state due to its geographic barriers. Data collection with an electronic survey had some bias due to the low rate of responses, the nonrepresentative nature of the studied population, and the self-selection of participants, also called the volunteer effect. Unfortunately, our study has a low rate of responses; the sampling was not probabilistic; therefore, it does not represent the population of Amazonian pediatricians. Another limitation is the lack of a validated instrument to assess knowledge and discomfort with EC prescription. Therefore, more studies should be conducted to better understand the associated factors with EC prescription discomfort among pediatricians. One exciting study design should be an all-over-country survey supported by the Brazilian Pediatric Society with probabilistic sampling.

On the contrary, despite these limitations, the present study produced important information about factors associated with discomfort with EC prescription among pediatricians using models based on artificial intelligence to improve our understanding of the subject. It was the first study conducted in Amazonas, Brazil.

This study supports medical teaching programs and public policies to reduce unplanned adolescent pregnancy. Medical education programs should include practical educational strategies focusing on medical skills for contraceptive counseling, such as SBME and objective structured clinical examinations. These techniques assess selected competencies and communication skills in standardized, simulated scenarios.^21^ The public health system and medical professional associations should also promote continuing education for health professionals and the community since knowledge and access to EC prevent unplanned pregnancies.^22,25^ Moreover, contraceptive counseling for adolescents is an essential skill for pediatricians.^13^ Therefore, regional and national medical residency programs in pediatrics should emphasize this subject in the program. The development of artificial intelligence tools to support clinical decision-making in contraceptive prescription is a promising field for research.^29^ Possibly, such instruments could reduce physicians’ discomfort with EC prescriptions.

In conclusion, EC is still little known and infrequently prescribed by pediatricians of Amazonas, Brazil. Most of them feel uncomfortable prescribing EC, leading to its underuse. Some factors associated with discomfort with EC prescription were previous EC prescription, inexperience, misconceptions, and belief that the EC could stimulate risky sexual behavior or compromise adherence to other contraceptive methods.

## Data Availability

The database that originated the article is available in an open repository (https://osf.io/fz2kd/?view_only=9c8d0d3de96a47e7a8be8ad0b972fe8d).
